# Inflammation Modulates RLIP76/RALBP1 Electrophile-Glutathione Conjugate Transporter and Housekeeping Genes in Human Blood-Brain Barrier Endothelial Cells

**DOI:** 10.1371/journal.pone.0139101

**Published:** 2015-09-25

**Authors:** Barbara Bennani-Baiti, Stefan Toegel, Helmut Viernstein, Ernst Urban, Christian R. Noe, Idriss M. Bennani-Baiti

**Affiliations:** 1 Department for Medicinal Chemistry, Institute of Pharmaceutical Chemistry, University of Vienna, Althanstrasse 14, 1090 Vienna, Austria; 2 Department of Biomedical Imaging and Image-guided Therapy, Vienna General Hospital (AKH), Medical University of Vienna, Waehringer-Guertel 18–20, 1090 Vienna, Austria; 3 Karl Chiari Lab for Orthopaedic Biology, Department of Orthopaedics, Medical University of Vienna, Waehringer Guertel 18–20, 1090 Vienna, Austria; 4 Department of Pharmaceutical Technology and Biopharmaceutics, University of Vienna, Althanstrasse 14, 1090 Vienna, Austria; 5 The B^2^ Scientific Group, Werdertorgasse 12/3/6, 1010 Vienna, Austria; Northwestern University, UNITED STATES

## Abstract

Endothelial cells are often present at inflammation sites. This is the case of endothelial cells of the blood-brain barrier (BBB) of patients afflicted with neurodegenerative disorders such as Alzheimer's, Parkinson's, or multiple sclerosis, as well as in cases of bacterial meningitis, trauma, or tumor-associated ischemia. Inflammation is a known modulator of gene expression through the activation of transcription factors, mostly NF-κB. RLIP76 (a.k.a. RALBP1), an ATP-dependent transporter of electrophile-glutathione conjugates, modulates BBB permeability through the regulation of tight junction function, cell adhesion, and exocytosis. Genes and pathways regulated by RLIP76 are transcriptional targets of tumor necrosis factor alpha (TNF-α) pro-inflammatory molecule, suggesting that RLIP76 may also be an inflammation target. To assess the effects of TNF-α on RLIP76, we faced the problem of choosing reference genes impervious to TNF-α. Since such genes were not known in human BBB endothelial cells, we subjected these to TNF-α, and measured by quantitative RT-PCR the expression of housekeeping genes commonly used as reference genes. We find most to be modulated, and analysis of several inflammation datasets as well as a metaanalysis of more than 5000 human tissue samples encompassing more than 300 cell types and diseases show that no single housekeeping gene may be used as a reference gene. Using three different algorithms, however, we uncovered a reference geneset impervious to TNF-α, and show for the first time that RLIP76 expression is induced by TNF-α and follows the induction kinetics of inflammation markers, suggesting that inflammation can influence RLIP76 expression at the BBB. We also show that MRP1 (a.k.a. ABCC1), another electrophile-glutathione transporter, is not modulated in the same cells and conditions, indicating that RLIP76 regulation by TNF-α is not a general property of glutathione transporters. The reference geneset uncovered herein should aid in future gene expression studies in inflammatory conditions of the BBB.

## Introduction

Endothelial cells line the lumen of the entire circulatory system including all blood and lymphatic vessels, myocardium, renal glomeruli, and the blood-brain barrier (BBB). Endothelial cells also infiltrate tumors during the process of neoangiogenesis, putting them at the center stage of virtually all sites of inflammation. Endothelial cells have thus been reported to be exposed to the pro-inflammatory tumor necrosis factor α (TNF-α) cytokine in acute systemic conditions such as inflammation in response to bacteremia [1, 2], acute localized inflammation following myocardial infarction [1, 3], ischemic stroke [4], angioplasty [5], or post-ischemic reperfusion injury [6, 7]. Endothelial cells are also subjected to pro-inflammatory signals in chronic conditions such as atherosclerosis [8–10], Crohn's disease and ulcerative colitis [11–13], multiple sclerosis [14], periodontitis [15–18], rheumatoid arthritis [19, 20], type 2 diabetes mellitus [21, 22], and several cancers [23, 24]. At the blood-brain barrier (BBB), endothelial cells can be exposed to inflammation associated with brain-specific injuries and disorders such as Alzheimer's disease [25], bacterial meningitis [26], brain edema due to head trauma [27], ischemia and hypoxia [28, 29], tumors [30], epilepsy [31, 32], multiple sclerosis [28, 29], or Parkinson's disease [33, 34]. Inflammation modulates gene expression in many tissues, including endothelial cells [24, 35–38], *via* mechanisms that involve activation of transcription factors, most importantly NF-κB [39–43].

Quantification of gene expression modulation is usually carried out by means of quantitative real-time reverse transcription-coupled PCR (qRT-PCR), and amplification data are normalized to one or more reference genes to account for intra- and inter-experimental variability. A reference gene needs to be expressed in the tissue considered, and ought not to be modulated by the physiological or pathological variables studied. Examples of good reference human genes include; *ACTB* and *GAPDH* in prostate cancer [44]; *GAPDH* and *YWHAZ* in idiopathic pregnancy-derived placenta [45]; and HPRT1 and SDHA in sepsis [46]. As may be noticed from the above examples, these reference genes are housekeeping genes. Not all housekeeping genes, however, are suitable as reference genes as exemplified by the analysis of 81 ribosomal protein (*Rpl*) genes in 22 different tissues [47]. Although all *Rpl* genes were expressed in all tissues tested, and as such qualify as housekeeping genes, none could be used as a reference gene owing to high expression variability. Thus, and as may be surmised from the list of tissues/reference genes above, a gene or set of genes may serve as a reference in one tissue but may be inadequate for another. It may be inferred from these studies, therefore, that reference genes and genesets need to be determined for each tissue and in each physiological or pathological setting. This is particularly important in inflammatory conditions whereby the expression of many housekeeping genes can be modulated [46, 48, 49].

Due to the importance of endothelial cell inflammation in the physiopathology of the BBB, and owing to the widespread use of human ECV304 endothelial cells in BBB models (e.g. [50–61]), we set out to identify reference genes in these cells in a BBB model following treatment with TNF-α. We also probed more than 300 cell and disease types for universal reference genes, and specifically queried inflammation datasets for inflammation-impervious reference genes. Finally, we researched the impact of inflammation on the RLIP76 and MRP1 electrophile-glutathione conjugate transporters in BBB endothelial cells and discuss the implications of the observed modulation in BBB inflammatory disorders.

## Materials & Methods

### Reagents

Iscove's modified Dulbecco's medium (IMDM), Ham's F-12, new born calf serum (NCS), L-glutamine, penicillin/streptomycin, and trypsin were obtained from Invitrogen Life Technologies (Gibco™, Carlsbad, CA, USA). Heparin was purchased from MP Biomedicals (Irvine, CA, USA). TNF-α (hBA-158, sc-4564) was obtained from Santa Cruz Biotechnology, Inc. (Santa Cruz, CA, USA). Primers for qRT-PCR experiments were obtained from Metabion (Martinsried, Germany), and Brilliant SYBR Green QPCR Master Mix was from Stratagene (La Jolla, CA, USA). All other materials, including amphotericin B, transferring, and gelatin were obtained from Sigma (St. Louis, MO, USA) unless otherwise specified.

### Cell Culture

Culture of human endothelial ECV304 cells in a BBB model was previously reported [53–56, 62]. For the purpose of this work, ECV304 cells were obtained from the European Collection of Cell Cultures (ECACC, Wiltshire, UK) and were cultured in C6 glial cells conditioned medium (C6CM). C6 cells were obtained from the German Cancer Research Center (DKFZ, Heidelberg, Germany) and were grown at 5% CO_2_ in 175 cm^2^ gelatin-coated tissue flasks at 37C in IF medium (1:1 mixture of IMDM and Ham's F-12), supplemented with 7.5% (v/v) NCS, 7 mM L-glutamine, 5 μg/ml transferrin, 0.5 U/ml heparin, 100 U/ml penicillin, 100 μg/ml streptomycin and 0.25 μg/ml amphotericin B. The supernatant of C6 cultures was collected every other day to yield the C6CM medium used to supplement the ECV304 cells. ECV304 cells were grown in IF medium/C6CM (1:1; v/v) at 37C in 5% CO_2_ for two weeks in six-well plates, then challenged with 5 ng/ml of TNF-α for 0, 2, 6, 24 or 48 hours prior to mRNA extraction.

### RNA isolation and cDNA synthesis

Total RNA extraction including on-column DNase I digestion was performed using the NucleoSpin RNA II Kit (Macherey-Nagel, Dueren, Germany). To evaluate RNA purity and yield, all RNA samples were assessed on the Agilent Bioanalyzer 2100 using Nano LabChip analysis (Agilent Technologies, Palo Alto, California). Only those samples with an RNA integrity number exceeding 7 were used for reverse transcription (RT). Reverse transcription to cDNA was performed using the High Capacity cDNA Reverse Transcription Kit (Applied Biosystems, Foster City, USA) following manufacturer's instructions. Briefly, 1 μg of total RNA was reverse-transcribed in a 20 μl reaction using oligo(dT) primers and 2 U/μl RNase inhibitor (Invitrogen, Carlsbad, USA). All cDNA preparations were diluted at a ratio of 1:5 with RNase-free water prior to quantitative PCR.

### Reference gene selection

To avoid any possible gene or species confusion [63], we list herein the NCBI Gene IDs of all tested genes. Nine housekeeping genes commonly used as reference genes were selected to evaluate their expression stability following treatment with TNF-α. These were *ACTB* (NCBI GeneID 60), *B2M* (NCBI GeneID 567), *GAPDH* (NCBI GeneID 2597), *HPRT1* (NCBI GeneID 3251), *PMM1* (NCBI GeneID 5372), *PSMB6* (NCBI GeneID 5694), *SDHA* (NCBI GeneID 6389), *TUBA1B* (NCBI GeneID 10376), and *YWHAZ* (NCBI GeneID 7534). In addition, we researched the expression of the *76-kDa Ral-interacting protein* (*RLIP76* a.k.a. *RALBP1*; NCBI GeneID 10928) and *multidrug resistance protein 1* (*MRP1* a.k.a. *ABCC1*, NCBI GeneID 4363) glutathione conjugate transporters, as well as the following inflammation markers: *intercellular adhesion molecule 1* (*ICAM1*; NCBI GeneID 3383), *vascular cell adhesion molecule 1* (*VCAM1*; NCBI GeneID 7412), and *monocyte chemoattractant protein-1* (*MCP1* a.k.a. *CCL2*; NCBI GeneID 6347).

### Quantitative real-time PCR of reverse transcription products (qRT-PCR)

Owing to the importance of transparent and comprehensive reporting of essential technical information in qPCR [64], we have followed the guidelines for the design and documentation of qPCR experiments as recently outlined [65], and provide a checklist detailing all information pertaining to the technical adequacy of used qPCR protocols (S1 Table).

All qRT-PCR reactions were performed in reaction mixtures containing 1 μl cDNA, 12.5 μl Brilliant SYBR Green QPCR Master Mix, primers, and nuclease-free water to 25 μl. Biological replicates were run in duplicate on an Mx3000P QPCR system (Stratagene La Jolla, CA, USA). Thermocycling conditions consisted of an initial polymerase activation step at 95°C for 10 min, followed by 45 cycles at 95°C for 30 sec, 55°C for 1 min, and 72°C for 1 min. Melting curves were generated in the range of 55 to 95°C to confirm single gene-specific peaks and to detect potential primer-dimer formation. No-template controls were included in each run to control for potential sample contaminations, and data were analyzed using the MxPro software v.4 (Stratagene). Baselines and thresholds were automatically set by the software and further tested through manual inspection. The crossing point of the amplification curve through the threshold represented the cycle threshold (Ct). Ct values were transformed to quantities based on the comparative Ct method that takes into consideration respective amplification efficiencies. Following appropriate formatting, data were imported into *geNorm* [66], *NormFinder* [67], and *Bestkeeper* [68] VBA algorithms.

### Bioinformatic and statistical analyses

To query the modulation of gene expression by inflammation in patients, we searched the GEO database (http://www.ncbi.nlm.nih.gov/gds) for inflammation-related datasets. Three datasets encompassed genes of interest and included probes that passed quality criteria as previously established [69, 70]. These allowed us to probe for gene expression differences between healthy individuals and patients suffering of inflammatory bowel diseases (ulcerative colitis and Crohn's disease; GDS1615), idiopathic inflammatory myopathy (dermatomyositis, polymyositis, and inclusion body myositis; GDS2153), or rheumatoid arthritis (GDS3192). To test for gene expression variability in a wide array of human tissues, diseases, and drug treatments, we run an expression profiling metaanalysis for these genes in 5372 human tissues (ArrayExpress; E-MTAB-62; [71]). Samples were clustered in 15 or 96 metagroups and median expression of genes within each metagroup was compared to median expression across all samples. Statistical analyses were conducted using two-tailed unpaired t tests or one-way ANOVA with post-hoc Tukey tests cross-comparing all study groups, as appropriate. Data were considered significant at p < 0.05.

## Results & Discussion

To identify suitable reference genes in endothelial cells of a human BBB model subjected to pro-inflammatory stimuli, we researched the gene expression stability, or lack thereof, of nine frequently used reference genes following treatment with TNF-α. These included β-actin (*ACTB*), β2-microglobulin (*B2M*), glyceraldehyde-3-phosphate dehydrogenase (*GAPDH*), hypoxanthine phosphoribosyltransferase 1 (*HPRT1*), phosphomannomutase 1 (*PMM1*), proteasome subunit Y (*PSMB6*), succinate dehydrogenase complex subunit A (*SDHA*), α-tubulin (*TUBA1B*) and tyrosine 3-monooxygenase/tryptophan 5-monooxygenase activation protein, zeta polypeptide (*YWHAZ*). The amplification profiles of each housekeeping gene were analyzed using three different algorithms implemented in the *BestKeeper*, *geNorm*, and *NormFinder* VBA applets. Once we identified a reference geneset, we used it as a Normalization Factor to assess expression modulation by TNF-α of additional test genes.

### TNF-α induces the expression of inflammation markers in BBB endothelial cells

**Fig 1:**
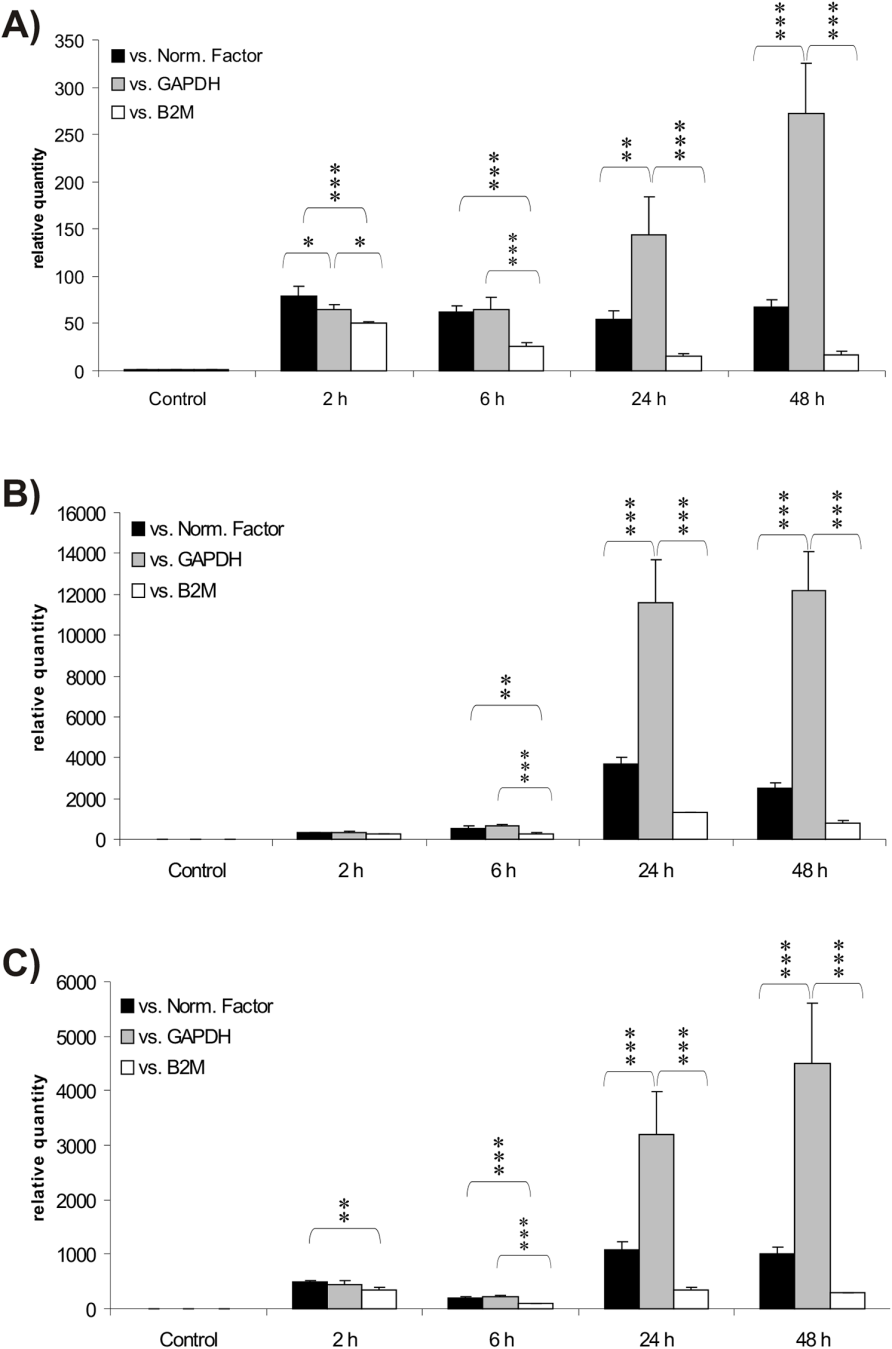
TNF-α induces the expression of inflammation markers in blood-brain barrier endothelial cells. Expression levels of *ICAM1* (Panel A), *VCAM1* (Panel B) and *MCP1* (Panel C) were monitored by qRT-PCR prior (control) and 2 to 48 hours following TNF-α treatment. Expression levels were normalized against *PSMB6* / *HPRT1* as a reference geneset (Normalization Factor; black graphs), or against single genes, here *GAPDH* (grey graphs) or *B2M* (white graphs). Results are presented as arithmetic means ± standard deviations. Statistical significance: * signifies p < 0.05, ** p < 0.01, and *** p < 0.001; lack of asterisks denotes lack of a statistically significant difference.

To test whether TNF-α treatment mimics inflammation in BBB endothelial cells, we carried out qRT-PCR experiments to follow the gene expression kinetics of endothelial/vascular inflammation markers. All three inflammation markers tested were strongly induced as early as 2 hours post-TNF-α treatment (Fig 1). Using the reference geneset identified below as a Normalization Factor, we find *ICAM1* induction to be maximal at 2 hours (Fig 1A), whereas both *VCAM1* and *MCP1* were maximally induced at 24 hours (Fig 1B and 1C). All three markers remained highly expressed for the duration of the experiment, indicating that the experimental conditions used were commensurate with an inflammation model. As shown in Fig 1A–1C, fold induction of the three genes was overly exaggerated, or instead underestimated, by use of single housekeeping genes as reference genes. The large variations observed in *ICAM1*, *VCAM1* and *MCP1* gene expression levels following normalization with single housekeeping genes underscore the need for a stably expressed reference geneset to normalizing gene expression data.

### Evaluation of candidate reference genes using *BestKeeper*

*BestKeeper* determines reference gene stability by employing pairwise correlation analysis of all candidate gene pairs and combines the best suited standards into a "*BestKeeper* index" [68]. A Pearson correlation coefficient (*r*) and probability value (*p*) are ascribed to each gene to describe the consistency between each candidate gene and the *BestKeeper* index. Using this algorithm, *B2M* exhibited a regulation inverse to that of the index, indicated by the negative correlation coefficient (*r* = -0.164). The other candidate genes showed a positive correlation with the index (Table 1). Descriptive statistics of the Ct values under the present experimental conditions, as computed by *BestKeeper*, are presented in Table 1. All Ct values were compared over the entire dataset, including control and treatment groups. An estimation of reference gene expression stability is provided by a comparison of standard deviations (SD values). Candidate reference genes ranking from the most to the least stable were *SDHA ≈ HPRT1 > PMM1 ≥ PSMB6 > TUBA1B > YWHAZ ≥ ACTB > B2M > GAPDH*. In this setup, *B2M* and *GAPDH* expression was found to be highly variable, arguing against using them as reference genes.

**Table 1:** Statistical output of the *Bestkeeper* analysis. Candidate reference genes are listed according to increasing standard deviations (SD). Consistency between each candidate gene and the *Bestkeeper* index is described by the Pearson correlation coefficient (*r*) and the probability value (*p*), based on all candidate genes (n = 9) or after exclusion of *GAPDH* and *B2M* (n = 7). Abbreviations: Ar. Mean: arithmetic mean; Geo. Mean: geometric mean; BK: *Bestkeeper* index.

Variable	SDHA	HPRT1	TUBA1B	PSMB6	ACTB	PPMM1	YWHAZ	B2M	GAPDH	BK
**n**	20	20	20	20	20	20	20	20	20	20
**Geo M.**	20.39	20.59	15.79	20.00	14.90	22.89	17.44	17.52	16.01	18.22
**Ar. M.**	20.39	20.59	15.79	20.00	14.91	22.90	17.45	17.54	16.04	18.22
**Min Ct**	19.88	20.13	15.27	19.19	13.91	22.35	16.55	16.49	14.64	17.63
**Max Ct**	20.83	21.23	16.56	20.91	15.56	23.62	18.52	18.97	18.01	18.77
**SD ± Ct**	0.21	0.22	0.31	0.33	0.36	0.36	0.41	0.67	0.93	0.26
**CV % Ct**	1.03	1.06	1.99	1.65	2.43	1.56	2.35	3.79	5.80	1.45
***r* (n = 9)**	0.305	0.919	0.654	0.862	0.869	0.745	0.925	-0.164	0.854	-
***p* (n = 9)**	0.191	0.001	0.002	0.001	0.001	0.001	0.001	0.487	0.001	-
***r* (n = 7)**	0.258	0.935	0.732	0.875	0.827	0.781	0.924	-	-	-
***p* (n = 7**)	0.273	0.001	0.001	0.001	0.001	0.001	0.001	-	-	-

### Assessment of candidate reference genes using *geNorm*

*geNorm* is based on the principle that the expression ratio of two ideal reference genes is constant in all samples, regardless of experimental conditions [66]. Table 2 shows the ranking of all candidate reference genes according to their stability measure M (average pairwise variations). Genes with the lowest M values are characterized by the most stable expression. According to the *geNorm* analysis, the three most stable reference genes across all samples and experimental conditions were *SDHA*, *PSMB6*, and *HPRT1*. The order of the top two genes could not be further ranked by *geNorm* due to the intrinsic requirement of gene ratios for gene stability ranking in this methodology. Again, in agreement with *BestKeeper* analysis, *B2M* and *GAPDH* proved to be the most variably expressed genes under the experimental conditions tested (Table 2).

**Table 2:** Candidate reference genes ranked according to *geNorm*. M values were calculated as average expression stability of reference genes during stepwise exclusion of the least stable control. V values were calculated as the pairwise variation between two sequential Normalization Factors.

Ranking	Gene	M value	V value
**1/2**	SDHA/PSMB6	0.24	-
**3**	HPRT1	0.27	0.086
**4**	PPMM1	0.29	0.069
**5**	TUBA1B	0.32	0.067
**6**	ACTB	0.35	0.061
**7**	YWHAZ	0.39	0.06
**8**	GAPDH	0.51	0.109
**9**	B2M	0.62	0.107

Choosing the appropriate number of reference genes for qRT-PCR normalization involves a compromise between practical considerations and accuracy. *geNorm* permits to calculate the pairwise variations V_n_/V_n+1_ between two sequential Normalization Factors (NF-_n_ and NF-_n+1_) to determine the added benefit of including additional reference genes for reliable normalization. *geNorm* thus allows to determine the minimum number of genes required to achieve the commonly accepted V-value threshold of 0.15, the maximum allowed for reference gene stability. In our study, the inclusion of a third reference gene yields a V-value V_2/3_ of 0.086 (significantly below the 0.15 cut-off value), indicating that two of the top three genes (*SDHA*, *PSMB6*, *HPRT1*) suffice as a Normalization Factor.

### Ranking of candidate reference genes using *NormFinder*

The ranking of candidate genes provided by *geNorm* was further tested by a *NormFinder* analysis (Table 3). The most notable differences in the *NormFinder* test were the ranking of *ACTB* as the most stable gene, and the ranking of *SDHA* near the bottom. Again, as in the previous two analyses, *GAPDH* and *B2M* proved to be the least stably expressed of all tested genes. An analysis of promoter sequences of all housekeeping genes revealed the presence of NF-κB binding sites in *GAPDH* and *B2M*, providing a possible molecular basis for their modulation by TNF-α (~2-fold overrepresentation compared to the average random promoter, z-scores > 1; Table 4). Interestingly, the ranking of genes based on the significance of NF-κB binding sites within promoter sequences (Table 4) was very similar to the ranking of gene expression stability under TNF-α obtained with the *NormFinder* algorithm (Table 3). Thus, based on the *NormFinder* ranking, gene loci not modulated by TNF-α had no significant hits for NF-κB binding sites, whereas those modulated by TNF-α had the most hits. The genome-wide comparative analysis of NF-κB binding sites summarized in Table 4 further shows that NF-κB binding sites are underrepresented in *PSMB6* and *HPRT1* gene promoters, both when compared to the average of all genome promoters, or to the other housekeeping genes tested individually. While these data are consistent with the lack of modulation of *PSMB6* and *HPRT1* by TNF-α, they may or not reflect the activity of NF-κB on these two genes *in vivo*, which remains to be ascertained. These considerations notwithstanding, since *PSMB6* and *HPRT1* were the two housekeeping genes most consistently impervious to TNF-α, we used their average expression as a Normalization Factor for the remaining of this study.

**Table 3:** Candidate reference genes ranked according to *NormFinder*. In this analysis, genes ranked 1–4 were deemed highly stable, whereas genes ranked 8–9 were deemed highly unstable.

Ranking	Gene	Stability value
**1**	ACTB	0.083
**2**	HPRT1	0.092
**3**	PSMB6	0.094
**4**	TUBA1B	0.107
**5**	PPMM1	0.175
**6**	YWHAZ	0.186
**7**	SDHA	0.209
**8**	B2M	0.730
**9**	GAPDH	0.845

**Table 4:** NF-κB binding sites within housekeeping gene promoters. 2000 bp of 5' and 500 bp of 3' flanking sequences were searched for NF-κB binding sites (GGGRNNYYCC; whereby R is a purine and Y is a pyrimidine) and compared to the average binding site distribution of similarly-sized random genomic or promoter sequences. *z-*statistics show that *B2M* and *GAPDH* promoters contain significantly more NF-κB binding sites than the other housekeeping genes or the average genomic or promoter sequence, whereas NF-κB binding sites are underrepresented in PSMB6 and HPRT1.

Reference Gene	# input sequences with match	# of matches in input	Expected in genome	SD	Over-representation vs genome	Z-score vs genome	Expected in promoters	SD	Over-representation vs promoters	Z-score vs promoters
**PSMB6**	1	1	2	1.41	0.5	-1.06	3.06	1.75	0.33	-1.46
**HPRT1**	1	2	1.96	1.4	1.02	-0.33	3	1.73	0.67	-0.87
**ACTB**	1	4	2.06	1.44	1.94	1.06	3.16	1.78	1.27	0.19
**TUBA1B**	1	4	2.03	1.41	1.97	1.06	3.12	1.73	1.28	0.21
**PMM1**	1	4	2.01	1.42	1.99	1.05	3.08	1.75	1.3	0.24
**YWHAZ**	1	5	2.05	1.43	2.44	1.71	3.13	1.77	1.6	0.77
**SDHA**	1	6	2.07	1.44	2.9	2.39	3.28	1.78	1.83	0.82
**GAPDH**	1	6	2.54	1.59	2.36	1.86	3.16	1.97	1.9	1.32
**B2M**	1	7	1.98	1.41	3.53	3.21	3.03	1.74	2.31	1.99

### Comparison of the reference genes identified herein to those of other studies

To the best of our knowledge, there are only a few reported studies wherein reference genes were investigated in model systems of human endothelial cells undergoing inflammation. Similarly to our finding that *HPRT1* is stably expressed in BBB endothelial cells, *HPRT1* is also the gene most commonly reported to be impervious to pro-inflammatory stimuli in endothelial cells, as recently reported for human endothelial cells of the umbilical vein, choroid, and retina [72–74]. *PSMB6*, our second component of the Normalization Factor was also proposed as a reference gene in umbilical vein endothelial cells [75]. Other genes found here to be expressed at relatively stable levels after TNF-α treatment were also proposed as inflammation-stable reference genes in endothelial cells of the umbilical vein (e.g. *ACTB* [75], and *YWHAZ* [72]), or in endothelial cells of the lungs (e.g. *TUBA1B* [76]). However, contrary to this last study which also proposed *GAPDH* as a reference gene, we find *GAPDH* to be one of the least reliable reference genes. The discrepancy between the results of the studies may be due to the difference of tissues tested or may be due to the different stimuli used, which among other things can determine tissue-specific chromatin accessibility and ensuing gene transcriptional permissiveness [77, 78]. This calls for a cautionary note concerning the use of genes reported as good references in studies using similar cells from different tissues, or that were subjected to different physical or humoral environments. This further argues in favor of using the average of several genes as a reference set, preferably in comparable cells and tissues subjected to similar stimuli.

### Lack of universal inflammation-impervious reference genes

**Fig 2:**
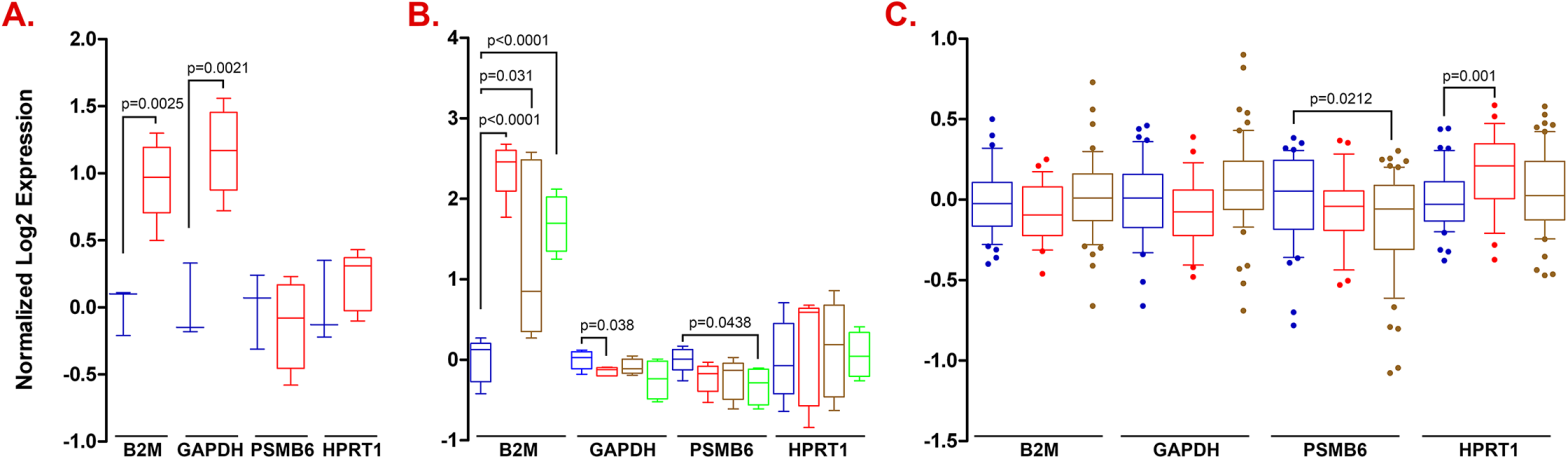
Modulation of housekeeping gene expression in several inflammatory conditions. **A.** Expression was monitored in synovial fluid macrophages from healthy individuals (blue) or patients suffering from rheumatoid arthritis (red); **B.** Expression was monitored in skeletal muscle fibers from healthy individuals (blue), dermatomyositis patients (red), polymyositis patients (gold), and inclusion body myositis patients (green); **C.** Expression was monitored in peripheral blood mononuclear cells from healthy individuals (blue), patients suffering from ulcerative colitis (red), or of Crohn's disease (gold).

To test whether genes uncovered here to be either impervious (e.g. *PSMB6* and *HPRT1*) or sensitive to inflammation stimuli (e.g. *B2M* and *GAPDH*) behave similarly in other inflammatory conditions, we analyzed gene expression in healthy individuals and patient populations suffering from one of several inflammatory conditions. Similarly to our findings in TNF-α treated endothelial cells, *PSMB6* expression was not modulated in rheumatoid arthritis (Fig 2A), dermatomyositis (Fig 2B), polymyositis (Fig 2B), and ulcerative colitis (Fig 2C), and *HPRT1* expression was stable in rheumatoid arthritis (Fig 2A), all tested idiopathic inflammatory myopathies (Fig 2B), and in ulcerative colitis (Fig 2C). Also akin to findings herein, *B2M* expression was highly modulated in rheumatoid arthritis (Fig 2A) and in all tested idiopathic inflammatory myopathies (Fig 2B), and *GAPDH* expression was modulated in rheumatoid arthritis and dermatomyositis (Fig 2C). However, in contrast to our findings for these genes in TNF-α treated endothelial cells, *PSMB6* was significantly modulated in inclusion body myositis (Fig 2B) and both *PSMB6* and *HPRT1* were modulated in Crohn's disease (Fig 2C). Moreover both *B2M* and *GAPDH* were stably expressed in inflammatory bowel diseases and *GAPDH* was in addition stably expressed in polymyositis and in inclusion body myositis (Fig 2C). These data show the limited "portability" of reference gene status, or lack thereof, from one inflammatory condition to another, which, therefore, ought to be independently determined for each tissue and pro-inflammatory stimulus involved.

### Lack of evidence supporting the concept of a universal reference gene or geneset

**Fig 3:**
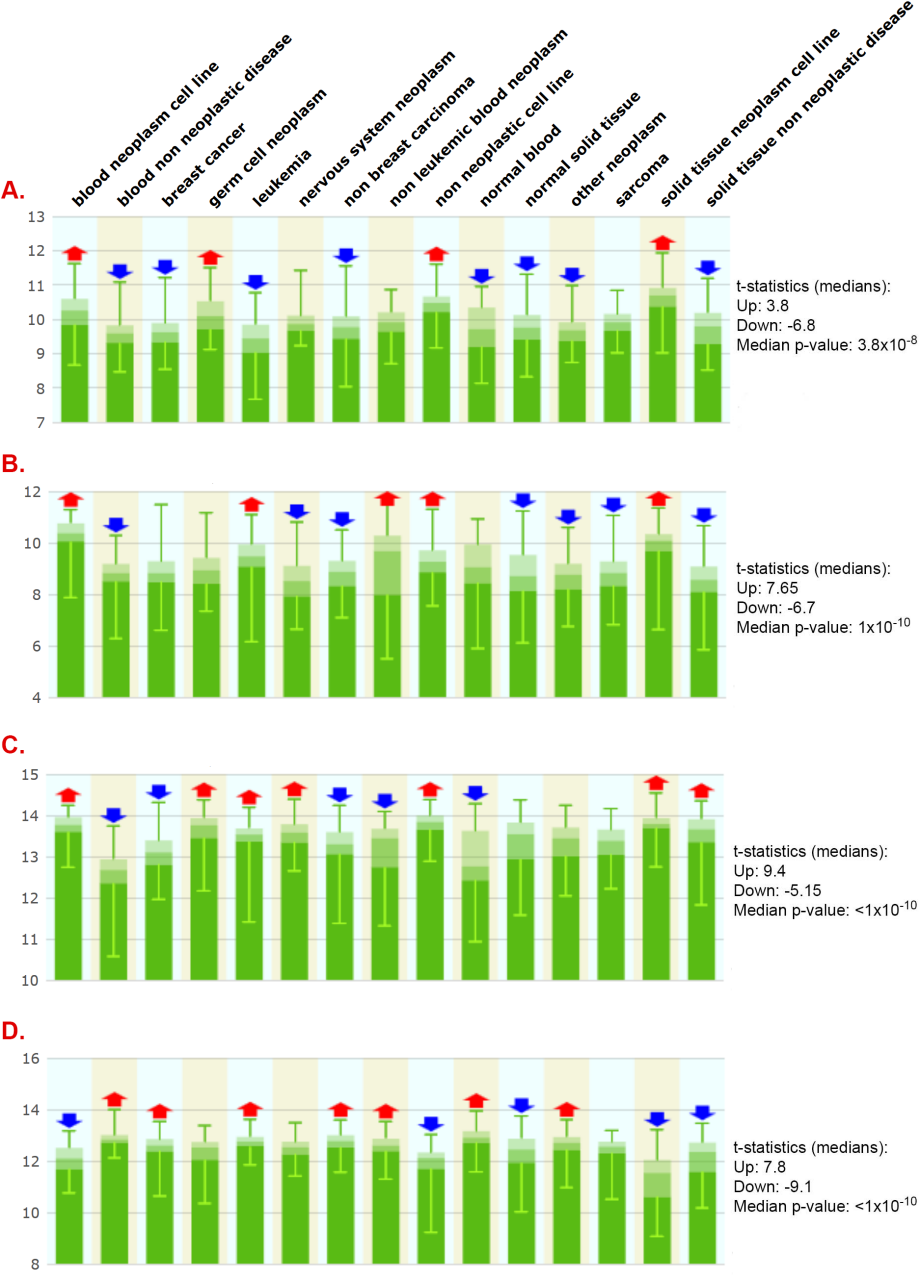
Gene expression variability of the PSMB6, HPRT1, B2M and GAPDH genes in human cells and diseases. Gene expression of PSMB6 (**A**), HPRT1 (B), GAPDH (C), and B2M (D) was assessed across 5372 human tissue samples representing 369 tissues and cell types or diseases clustered into 15 meta-groups (indicated on top of figure). Red and blue arrows indicate expression significantly above (Up) or below (Down) median expression across 15 meta-groups, respectively. Medians of t-statistics and associated p-values are indicated. Ordinate axis: Log2 expression.

**Fig 4:**
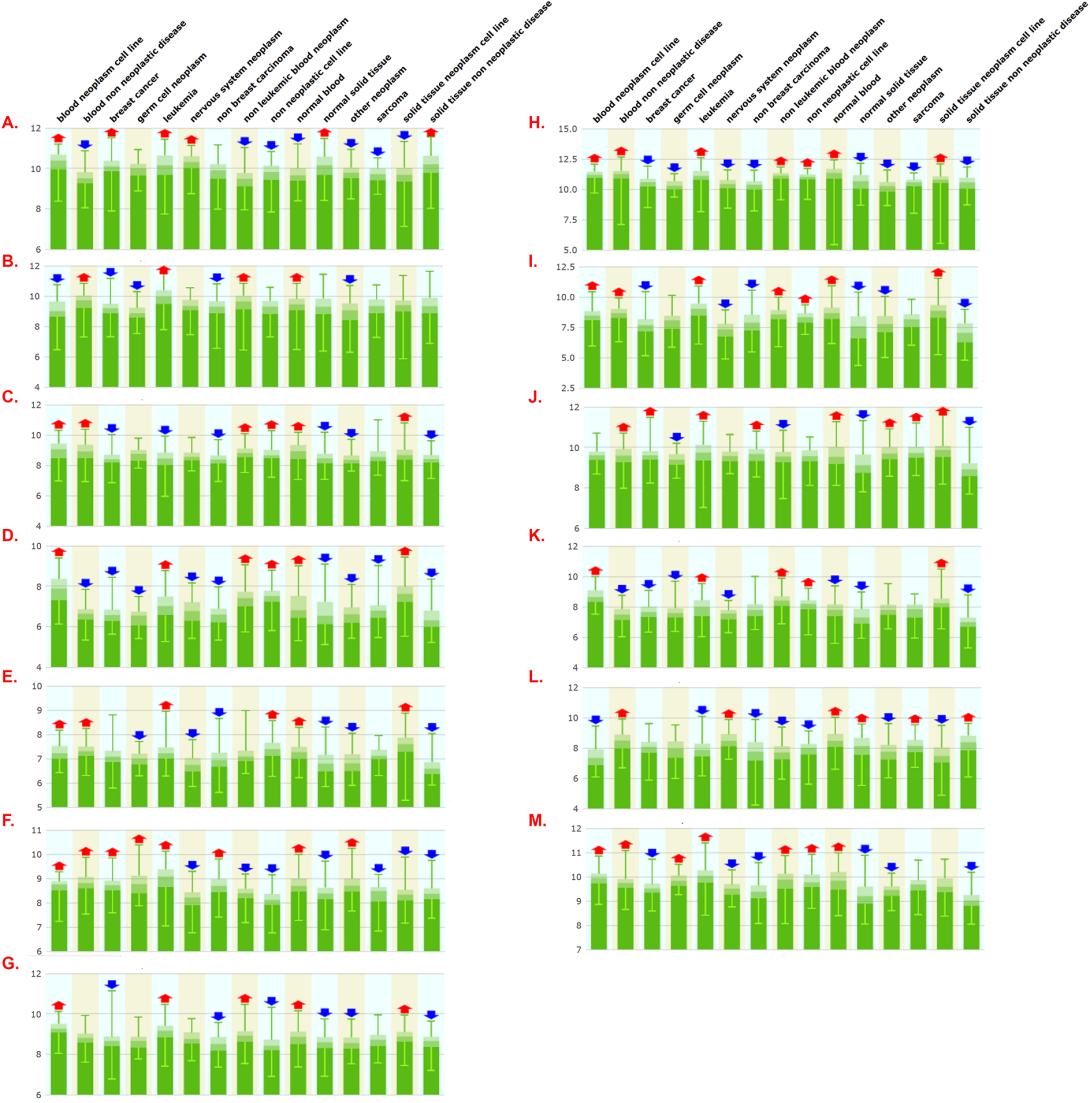
Gene expression variability of 'universal reference genes' in human cells and diseases. Gene expression of previously reported universally stably expressed reference genes was monitored across 5372 human tissues samples representing 369 tissues and cell types or diseases clustered into 15 meta-groups (indicated on top of figure). Red and blue arrows indicate expression significantly above (Up) or below (Down) median expression within meta-group, respectively. p-values ranged from 10^−2^ to less than 10^−10^. **Panels: A.** ARL8B; **B.** CTBP1; **C.** CUL1; **D.** DIMT1L; **E.** FBXW2; **F.** GPBP1; **G.** LUC7L2; **H.** OAZ1; **I.** PAPOLA; **J.** SPG21; **K.** TRIM27; **L.** UBQLN1; **M.** ZNF-207.

To further assess *PSMB6*, *HPRT1*, *B2M*, and *GAPDH* gene expression stability, we run a multivariate expression analysis encompassing 5,372 human tissue samples and 369 tissue and disease types. As shown in Fig 3, *PSMB6* was again the least variable gene, followed by *HPRT1* and *B2M/GAPDH*. However, as shown in Fig 3 and detailed in S2–S5 Tables, expression of all genes was highly variable in most metagroups, indicating that no gene is expressed stably enough to serve as a single reference gene in most cell types and conditions. To further test this premise, we also analyzed expression of 13 previously reported reference genes proposed to be stably expressed across different tissues based on *in silico* and molecular analyses [79]. As shown in Fig 4, all 13 genes show high gene expression variability in most 15 metagroups. Detailed analysis of the number of experiments wherein gene expression varies across the 369 tissue, cell, and disease types shows *HPRT1* and *SPG21* to be the least variably expressed genes (Table 5). However, cluster analysis in 96 metagroups shows *SPG21* and *HPRT1* expression to nonetheless significantly vary in 70 and 81 out of 96 metagroups, respectively (S6 and S7 Tables). Together, these data strongly support the need to validate reference genes in most tissues and conditions.

**Table 5:** Modulation of gene expression by drugs or disease state across several tissue and cell types. Expression of 13 previously reported 'universal reference genes' and of the most and least stably expressed genes uncovered in this study was monitored across 5372 human samples representing 369 different cell and tissue types, disease states and cell lines. Data show the number of studies documenting gene expression modulation for the considered parameters.

Gene	Experiments	Cell types	Cell lines	Diseases	Compound treatment
**ARL8B**	306	85	307	84	168
**CTBP1**	343	90	301	99	315
**CUL1**	297	71	213	77	114
**DIMT1L**	364	96	318	102	208
**FBXW2**	242	67	256	51	180
**GPBP1**	302	87	272	87	180
**LUC7L2**	268	80	295	74	77
**OAZ1**	284	93	194	82	199
**PAPOLA**	427	125	392	135	315
**SPG21**	243	72	226	67	101
**TRIM27**	383	107	329	105	129
**UBQLN1**	296	85	217	74	160
**ZNF207**	420	124	365	136	310
**HPRT1**	334	94	264	83	115
**PSMB6**	267	68	222	63	155
**B2M**	319	102	285	94	175
**GAPDH**	334	108	227	102	93

### Expression of *RLIP76*, but not of *MRP1*, is modulated by TNF-α

*RLIP76* is a tightly regulated gene expressed in all human tissues and cell lines so far examined. The *RLIP76* gene encodes a GTPase-activating protein and a downstream effector of the RALA and RALB ras-like GTP-binding proteins, thus modulating the mitotic spindle, clathrin-dependent endocytosis, tight junctions, cell-to-cell adhesion, targeting of proteins to basolateral plasma membranes, and the assembly of exocyst complexes and secretagogue-dependent exocytosis [80–82]. RLIP76 was also recently shown to interact with ARNO (a.k.a. Cytohesin 2), a guanine-nucleotide exchange Sec7 domain-containing protein that works in concert with ARF6 (ADP-ribosylation factor 6), a small guanine nucleotide-binding protein of the RAS superfamily [83]. RLIP76 activates RAC1 in an ARF6- and PI3K-dependent manner, and regulates also cell migration and spreading through the activities of ARNO and ARF6 [83]. In addition, RLIP76 regulates HIF1α (Hypoxia Inducible Factor 1, alpha subunit) and VEGF (Vascular Endothelial Growth Factor), and is essential to proper endothelial cell function, normal angiogenesis, and to tumor-associated neoangiogenesis [84, 85]. Finally, RLIP76 is also a non-ATP binding cassette drug transporter that effects the efflux of glutathione conjugates of electrophiles including those of xenobiotics, thus mediating drug resistance (e.g. of doxorubicin, vinorelbine, unitinib, sorafenib) in several mouse models bearing human cancer xenografts of the lungs, kidney, skin, colon, and prostate [86–93]. Owing to its expression at the BBB, RLIP76 can also lead to drug resistance of brain tumors, as well as in neurological disorders such as epilepsy (e.g. to phenytoin and carbamazepine) [86, 94].

**Fig 5:**
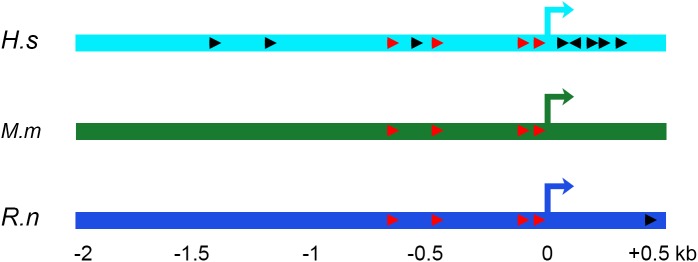
Comparative analysis of human, murine and rat *RLIP76* promoter sequences unveils conserved NF-κB binding sites. Human (*Homo sapiens*: *H*.*s*), murine (*Mus musculus*: *M*.*m*) and rat (*Rattus norvegicus*: *R*.*n*) DNA sequences spanning -2 to +0.5 kb relative to the transcription start site (+0 kb) were retrieved using the Cold Spring Harbor Laboratory promoter database (http://rulai.cshl.edu/cgi-bin/CSHLmpd2/promExtract.pl?). Sequence alignments were performed using NCBI Blast platform (http://blast.ncbi.nlm.nih.gov/Blast.cgi), and GGGRNNYYCC (whereby R is a purine and Y is a pyrimidine) was used as the consensus NF-κB binding site. Orientation of the binding sites is shown with arrow heads, with read arrowheads corresponding to evolutionarily conserved and black arrowheads non-conserved binding sites.

Since the BBB is often subjected to inflammation in brain disorders [32], and TNF-α is a known modulator of several RLIP76 downstream targets through the NF-κB transcription factor [81, 95–97], we asked whether TNF-α might also regulate *RLIP76*. A comparative analysis of human, murine and rat *RLIP76* 5' flanking sequences uncovered several evolutionarily conserved and non-conserved putative NF-κB transcription factor binding sites (Fig 5). Non-conserved binding sites mapped mostly to transcription start site (TSS)-distant upstream sequences and TSS-downstream sequences, whereas evolutionarily conserved NF-κB binding clustered within less than a kilobase upstream of the TSS. Since conservation of transcription factor binding sites across divergent species is an indication of regulatory element functionality [98, 99], the data suggest that *RLIP76* can be a target of pro-inflammatory molecules *via* a cluster of evolutionarily conserved NF-κB binding sites upstream of the TSS (Fig 5).

**Fig 6:**
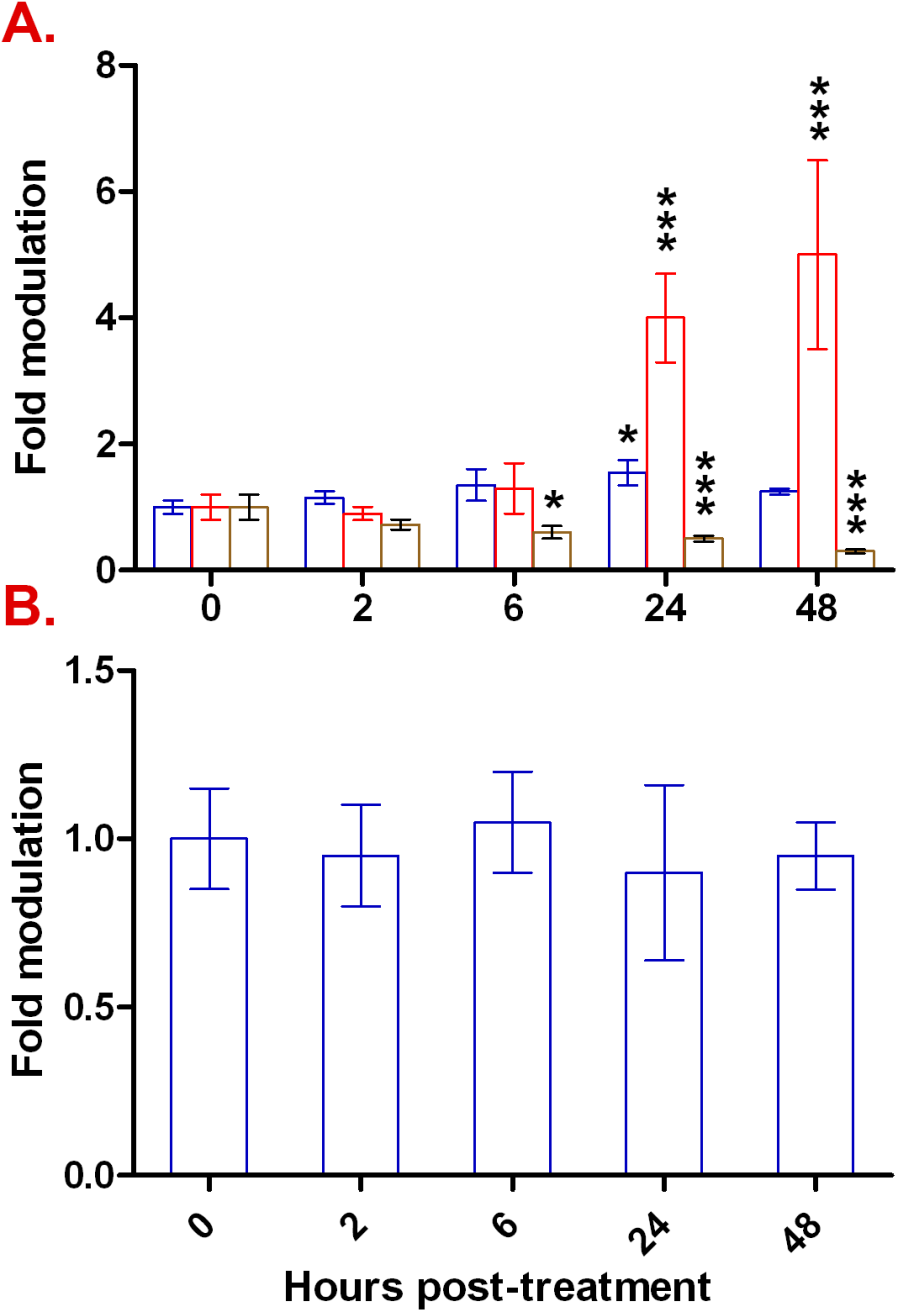
TNF-α induces *RLIP76* but not *MRP1* gene expression. Expression levels of *RLIP76* (Panel A) and *MRP1* (Panel B) were monitored by qRT-PCR prior and after TNF-α treatment for 2–48 hours relative to the *PSMB6/HPRT1* reference geneset (blue bars), *GAPDH* (red bars), or to *B2M* (brown bars). Results are presented as arithmetic means ± standard deviations. Statistical significance: * signifies p < 0.05, *** signifies p < 0.001; lack of asterisks denotes lack of a statistically significant difference.

We therefore sought to explore the effects of TNF-α on *RLIP76* gene expression in BBB endothelial cells. As shown in Fig 6A, TNF-α led to a gradual increase in *RLIP76* expression, reaching a maximum at 24 hours. To assess whether TNF-α modulation of *RLIP76* extends to other glutathione transporter-encoding genes, we also analyzed expression of the *MRP1* gene. As shown in Fig 6B, TNF-α had no measurable effect on *MRP1*, supporting the specificity of *RLIP76* modulation by TNF-α.

Normalization of the *RLIP76* expression data with single housekeeping genes, instead of the reference geneset, yielded very different and contrasting pictures, with either a maximal 500% induction of *RLIP76* (*GAPDH* as reference gene; Fig 6A), or 70% suppression of its expression (*B2M* as a reference gene; Fig 6A). Although not as dramatic, one may also see the effect of using inappropriate single housekeeping genes in the normalization of highly inducible genes. For instance, normalization of *ICAM1* using *GAPDH* would make its expression appear to gradually increase over the duration of the experiment by an additional 4–5 fold after the initial induction at 2h, thus giving the impression that *ICAM1* maximal induction is reached only 48h post-treatment (Fig 1A). On the opposite end of the spectrum, normalization against *B2M* expression would intimate the notion of an escape mechanism from TNF-α induction taking place sometime between 2 and 6 hours post-induction (Fig 1A). Using the Normalization Factor, however, we show *ICAM1* expression to be maximally induced 2h post TNF-α-treatment, and that the TNF-α-mediated induction is maintained at this level for the duration of the experiment (Fig 1A), consistent with previously reported *ICAM1* induction kinetics in endothelial cells subjected to pro-inflammatory stimuli [100, 101]. Together, and as may be surmised from a comparison of data in Figs 1 and 6 in which TNF-α either appeared to cause profound effects (Fig 1A–1C) or exerted modest gene modulation (Fig 6A), data normalization skewing was more pronounced in datasets in which gene expression modulation was moderate (for example here *RLIP76*). We submit, therefore, that it is particularly important to use appropriate reference genesets instead of single untested housekeeping genes when quantifying moderate gene expression variations.

The fact that RLIP76 is induced by inflammation in BBB endothelial cells can be relevant to central nervous system diseases with an inflammatory component. This is for instance the case of epilepsy [102–105], and drugs used to treat epilepsy such as carbamazepine and phenytoin [106] are RLIP76 substrates [86]. A corollary to these notions is whether inflammation can modulate anti-epileptic drug efficacy, and importantly, whether non-steroidal anti-inflammatory drugs (NSAIDs) can improve epilepsy treatment. While data in patients are still lacking, several FDA-approved NSAIDs including acetylsalicylic acid, ibuprofen, indomethacin, metamizole, paracetamol, and piroxicam were reported to augment the antiepileptic effectiveness of phenytoin in a mouse seizure model [107]. Assuming this model can be translated in a clinical setting in patients, it suggests that NSAIDs may be useful not only in the treatment of epilepsy-associated inflammation, but may in addition potentiate the efficacy of anti-epileptic drugs. Since RLIP76 expression is increased in the brain of carbamazepine- and phenytoin-refractory epileptic patients wherein it extrudes these drugs [86], and since inflammation often associates with epilepsy [102–105] and may increase RLIP76 expression (this report), it may be pertinent to explore whether NSAIDs ought to systematically be co-administered with anti-epileptic drugs in cases wherein drug resistance is documented. Finally, from a mechanistic perspective, it would be of interest to explore whether the potentiating effect of NSAIDs on anti-epileptic drugs is due to the down-modulation of RLIP76 gene expression.

## Conclusions

Treatment of human endothelial cells in a BBB model with TNF-α led to the induction of several inflammation markers indicating an inflammatory response. Under these conditions, the expression of housekeeping genes such as *HPRT1* and *PSMB6* remained relatively unaffected, whereas others showed time-dependent induction or suppression. Using the appropriate reference geneset, we show the *RLIP76* drug transporter to be induced by TNF-α. This induction is not a general property of all glutathione transporter-encoding genes as expression of *MRP1* was unchanged in the same conditions. Modulation of *RLIP76* by TNF-α might be relevant to the treatment outcome of brain disorders and tumors wherein the blood-brain barrier is inflamed, as it may alter drug transport across the barrier. Finally, we show that no gene or geneset may act as a universal reference gene, which therefore ought to be established for each cell type and associated pathophysiological condition.

## Supporting Information

S1 Table qRT-PCR technical checklist. Listed are the *in silico* and experimental variables pertaining to the design and conducting of qRT-PCR procedures on all genes tested.
(PDF)

S2 Table t-statics of PSMB6 gene expression variability in 5372 human tissue samples encompassing 369 tissue and disease types clustered in 15 metagroups. UP: up-regulated; DOWN: down-regulated; NONDE: non-differentially expressed. │t-statistic│> 2 were found to be significant (p<0.05).
(PDF)

S3 Table t-statics of HPRT1 gene expression variability in 5372 human tissue samples encompassing 369 tissue and disease types clustered in 15 metagroups. UP: up-regulated; DOWN: down-regulated; NONDE: non-differentially expressed. │t-statistic│> 2 were found to be significant (p<0.05).
(PDF)

S4 Table t-statics of GAPDH gene expression variability in 5372 human tissue samples encompassing 369 tissue and disease types clustered in 15 metagroups. UP: up-regulated; DOWN: down-regulated; NONDE: non-differentially expressed. │t-statistic│> 2 were found to be significant (p<0.05).
(PDF)

S5 Table t-statics of B2M gene expression variability in 5372 human tissue samples encompassing 369 tissue and disease types clustered in 15 metagroups. UP: up-regulated; DOWN: down-regulated; NONDE: non-differentially expressed. │t-statistic│> 2 were found to be significant (p<0.05).
(PDF)

S6 Table Expression of the SPG21 gene in 96 meta-groups encompassing 5372 human tissues samples representing 369 tissues, cell types, and diseases. Each metagroup contained at least 10 biological replicates. UP: up-regulated; DOWN: down-regulated; NONDE: non-differentially expressed. │t-statistic│ > 2 was found to be significant (p<0.05).
(PDF)

S7 Table Expression of the PSMB6 gene in 96 meta-groups encompassing 5372 human tissues samples representing 369 tissues, cell types, and diseases. Each metagroup contained at least 10 biological replicates. UP: up-regulated; DOWN: down-regulated; NONDE: non-differentially expressed. │t-statistic│ > 2 was found to be significant (p<0.05).
(PDF)
