# Supplementary material for: Inflammation Modulates RLIP76/RALBP1 Electrophile-Glutathione Conjugate Transporter and Housekeeping Genes in Human Blood-Brain Barrier Endothelial Cells
Source: PLoS One. 2015 Sep 25;10(9):e0139101. doi: 10.1371/journal.pone.0139101 (PMC4583384; doi:10.1371/journal.pone.0139101)
Supplement: S1 Table — Listed are the in silico and experimental variables pertaining to the design and conducting of qRT-PCR procedures on all genes tested. (PDF) [file pone.0139101.s001.pdf]

Sample/Template	details
Source	Human endothelial ECV304 cells in a blood-brain barrier model
Method of preservation	No preservation; Total RNA was extracted directly from PBS washed cell layers
Storage time (if appropriate)	RNA was stored at -80°C and reverse transcribed into cDNA within one month. cDNA was stored at -80°C and analyzed within 6 months.
Handling	----
Extraction method	Column based extraction method (NucleoSpin RNA II Kit (Macherey-Nagel, D).
RNA: DNA-free	All primer pairs used are intron-spanning primers. The RNA extraction protocol included on-column DNA digestion. Using microfluidics (Bioanalyzer), the quality of each RNA preparation was verified.
Concentration	All RNA preparations were quantified using microfluidics (Bioanalyzer).
RNA: integrity	Integrity of each RNA preparation was verified using microfluidics (Bioanalyzer).
Inhibition-free	Not all samples were tested for the absence of inhibitors. However, a representative set of samples was used to control for the absence of inhibitors using dilution series of target genes.
**Assay optimisation/validation**	
Accession numbers	
ACTB	NM_001101
B2M	NM_004048
GAPDH	NM_002046
HPRT1	NM_000194
ICAM1	NM_000201
MCP1	NM_002982
MRP1	NM_004996
PMM1	NM_002676
PSMB6	NM_002798
RLIP76	NM_006788
SDHA	NM_004168
TUBA1B	NM_006082
VCAM1	NM_001078
YWHAZ	NM_003406
Amplicon details	
ACTB	140 bp
B2M	86 bp
GAPDH	165 bp
HPRT1	94 bp
ICAM1	111 bp
MCP1	131 bp
MRP1	97 bp
PMM1	122 bp
PSMB6	82 bp
RLIP76	104 bp
SDHA	86 bp
TUBA1B	181 bp
VCAM1	87 bp
YWHAZ	94 bp

Primer sequence	
	**forward**
ACTB	CTGGAACGGTGAAGGTGACA
B2M	TGCTGTCTCCATGTTTGATGTATCT
GAPDH	GGAGTCCACTGGCGTCTTCAC
HPRT1	TGACACTGGCAAAACAATGCA
ICAM1	CCTATGGCAACGACTCCTTC
MCP1	AACACTGCCCTCCATCTAAAG
MRP1	GCTGATGGAGGCTGACAAG
PMM1	AAGCGTGGAACCTTCATCGA
PSMB6	ACCTGATGGCGGGAATCAT
RLIP76	AAGGCATCTACAGAGTATCAGG
SDHA	TGGGAACAAGAGGGCATCTG
TUBA1B	CTTTGAGCCAGCCAACCAGATG
VCAM1	CCTCCTTAATAATACCTGCCATTG
YWHAZ	ACTTTTGGTACATTGTGGCTTCAA
	**reverse**
ACTB	AAGGGACTTCCTGTAACAATGCA
B2M	TCTCTGCTCCCCACCTCTAAGT
GAPDH	GAGGCATTGCTGATGATCTTGAGG
HPRT1	GGTCCTTTTCACCAGCAAGCT
ICAM1	TCTCCTGGCTCTGGTTCC
MCP1	TCCACGACCTCCACTTCC
MRP1	GCTGAGGAAGGAGATGAAGAG
PMM1	TCCCGGATCTTCTCTTTCTTGTC
PSMB6	ATCATACCCCCCATAGGCACT
RLIP76	AGTGTTAGGCTCATAGTCTTCC
SDHA	CCACCACTGCATCAAATTCATG
TUBA1B	GATGCCAACCTTGAAGCCAGTG
VCAM1	GTGCTTCTACAAGACTATATGACC
YWHAZ	CCGCCAGGACAAACCAGTAT
*Probe sequence**	no probes used
In silico	All primers were subjected to BLAST analysis
empirical	Forward and reverse primers were used at the following concentrations (fwd/rev)
ACTB	300/100
B2M	300/900
GAPDH	300/600
HPRT1	300/900
ICAM1	100/100
MCP1	100/100
MRP1	100/100
PMM1	100/100
PSMB6	100/100
RLIP76	100/100
SDHA	300/900
TUBA1B	100/100
VCAM1	100/100
YWHAZ	600/600
Priming conditions	For cDNA synthesis, random RT primers were used as provided by the manufacturer (High Capacity cDNA Reverse Transcription Kit (Applied Biosystems, Austria)).
PCR efficiency	qPCR efficiencies were determined using dilution series of cDNA prepared from endothelial ECV304 cell mRNA. Used efficiencies are given below in %.
ACTB	97.0
B2M	100.5
GAPDH	97.5
HPRT1	96.5
ICAM1	94.0
MCP1	110.0
MRP1	94.5

PMM1	109.0
PSMB6	99.0
RLIP76	101.5
SDHA	100.0
TUBA1B	97.5
VCAM1	108.5
YWHAZ	99.0
Linear dynamic range	The dynamic range of the primers was spanning unknown targets as determined by dilution curves.
Limits of detection	LODs were not determined in this study.
Intra-assay variation	The intra-assay variation was below one Cq.
**RT/PCR**	
Protocols	**RT:** equal RNA quantities were reverse transcribed into cDNA using the High Capacity cDNA Reverse Transcription Kit (Applied Biosystems, Austria).<br>**qPCR:** reactions were performed in 25 µl reaction mixtures containing 1 µl cDNA, 12.5 µl Brilliant SYBR Green QPCR Master Mix (Stratagene, La Jolla, CA, USA), primers as indicated (Metabion, Martinsried, D), and nuclease-free water to 25 µl, and run in duplicate on a Mx3000P QPCR system (Stratagene La Jolla, CA, USA).
Reagents	see above.
Duplicate RT	Replicates of the RT step were not performed in this study.
NTC	NTCs were included in each run. The absence of both amplification and melting curve peaks were mandatory for the acceptance of the run.
NAC	Minus RT controls were not performed in this study.
Positive control	Inter-run calibrators were not used.
**Data analysis**	
Specialist software	Data were analyzed using the MxPro software v.4 (Stratagene) considering both amplification efficiencies and normalization to single reference genes or Normalization Factor.
Statistical justification	All qPCR reactions were performed in duplicate. Statistical analyses were conducted using two-tailed unpaired t tests or one-way ANOVA with post-hoc Tukey tests cross-comparing all study groups, as appropriate. Data were considered significant at $p < 0.05$.
Transparent, validated normalisation	The expression stability of 9 candidate reference genes was determined across the samples using geNorm, NormFinder, and BestKeeper VBA algorithms. Thus, PSMB6 and HPRT1 were selected as stable reference genes under the experimental conditions of this study and their average expression was used as a Normalization Factor.

Table S1.
