# Supplementary material for: Inflammation Modulates RLIP76/RALBP1 Electrophile-Glutathione Conjugate Transporter and Housekeeping Genes in Human Blood-Brain Barrier Endothelial Cells
Source: PLoS One. 2015 Sep 25;10(9):e0139101. doi: 10.1371/journal.pone.0139101 (PMC4583384; doi:10.1371/journal.pone.0139101)
Supplement: S2 Table — UP: up-regulated; DOWN: down-regulated; NONDE: non-differentially expressed. │t-statistic│> 2 were found to be significant (p<0.05). (PDF) [file pone.0139101.s002.pdf]

Metagroup	UP/DOWN	t-statistic	p-value
solid tissue neoplasm cell line	UP	42	1.00E-10
non neoplastic cell line	UP	19	1.00E-10
blood neoplasm cell line	UP	8	1.00E-10
germ cell neoplasm	UP	3.8	2.08E-04
non leukemic blood neoplasm	NONDE	0.43	6.98E-01
sarcoma	NONDE	0.33	7.68E-01
nervous system neoplasm	NONDE	0.24	8.28E-01
solid tissue non neoplastic disease	DOWN	-5.2	3.17E-07
non breast carcinoma	DOWN	-5.3	2.40E-07
other neoplasm	DOWN	-5.6	3.81E-08
normal solid tissue	DOWN	-5.6	3.93E-08
normal blood	DOWN	-8	1.00E-10
blood non neoplastic disease	DOWN	-13	1.00E-10
breast cancer	DOWN	-13	1.00E-10
leukemia	DOWN	-22	1.00E-10
