# Supplementary material for: Inflammation Modulates RLIP76/RALBP1 Electrophile-Glutathione Conjugate Transporter and Housekeeping Genes in Human Blood-Brain Barrier Endothelial Cells
Source: PLoS One. 2015 Sep 25;10(9):e0139101. doi: 10.1371/journal.pone.0139101 (PMC4583384; doi:10.1371/journal.pone.0139101)
Supplement: S3 Table — UP: up-regulated; DOWN: down-regulated; NONDE: non-differentially expressed. │t-statistic│> 2 were found to be significant (p<0.05). (PDF) [file pone.0139101.s003.pdf]

Metagroup	UP/DOWN	t-statistic	p-value
solid tissue neoplasm cell line	UP	28	1.00E-10
blood neoplasm cell line	UP	20	1.00E-10
leukemia	UP	10	1.00E-10
non neoplastic cell line	UP	5.3	2.66E-07
non leukemic blood neoplasm	UP	2.9	0.005
normal blood	NONDE	0.67	0.538
germ cell neoplasm	NONDE	-1.4	0.174
sarcoma	DOWN	-3.5	6.44E-04
non breast carcinoma	DOWN	-5.6	3.58E-08
other neoplasm	DOWN	-6.1	2.86E-09
blood non neoplastic disease	DOWN	-6.7	1.00E-10
breast cancer	DOWN	-6.9	1.00E-10
nervous system neoplasm	DOWN	-7.3	1.00E-10
normal solid tissue	DOWN	-10	1.00E-10
solid tissue non neoplastic disease	DOWN	-11	1.00E-10
