# Supplementary material for: Inflammation Modulates RLIP76/RALBP1 Electrophile-Glutathione Conjugate Transporter and Housekeeping Genes in Human Blood-Brain Barrier Endothelial Cells
Source: PLoS One. 2015 Sep 25;10(9):e0139101. doi: 10.1371/journal.pone.0139101 (PMC4583384; doi:10.1371/journal.pone.0139101)
Supplement: S4 Table — UP: up-regulated; DOWN: down-regulated; NONDE: non-differentially expressed. │t-statistic│> 2 were found to be significant (p<0.05). (PDF) [file pone.0139101.s004.pdf]

Metagroup	UP/DOWN	t-statistic	p-value
solid tissue neoplasm cell line	UP	23	1.00E-10
non neoplastic cell line	UP	15	1.00E-10
blood neoplasm cell line	UP	10	1.00E-10
solid tissue non neoplastic disease	UP	9.4	1.00E-10
leukemia	UP	6.6	1.00E-10
germ cell neoplasm	UP	4.3	2.54E-05
nervous system neoplasm	UP	3.5	7.59E-04
normal solid tissue	NONDE	-0.78	0.471
other neoplasm	NONDE	-1	0.351
sarcoma	NONDE	-1.5	0.165
non breast carcinoma	DOWN	-3.5	6.68E-04
non leukemic blood neoplasm	DOWN	-6.8	1.00E-10
breast cancer	DOWN	-15	1.00E-10
normal blood	DOWN	-21	1.00E-10
blood non neoplastic disease	DOWN	-34	1.00E-10
