# Supplementary material for: Inflammation Modulates RLIP76/RALBP1 Electrophile-Glutathione Conjugate Transporter and Housekeeping Genes in Human Blood-Brain Barrier Endothelial Cells
Source: PLoS One. 2015 Sep 25;10(9):e0139101. doi: 10.1371/journal.pone.0139101 (PMC4583384; doi:10.1371/journal.pone.0139101)
Supplement: S5 Table — UP: up-regulated; DOWN: down-regulated; NONDE: non-differentially expressed. │t-statistic│> 2 were found to be significant (p<0.05). (PDF) [file pone.0139101.s005.pdf]

Metagroup	UP/DOWN	t-statistic	p-value
normal blood	UP	19	1.00E-10
blood non neoplastic disease	UP	16	1.00E-10
leukemia	UP	14	1.00E-10
non breast carcinoma	UP	11	1.00E-10
breast cancer	UP	7.8	1.00E-10
non leukemic blood neoplasm	UP	7	1.00E-10
other neoplasm	UP	5.9	9.56E-09
nervous system neoplasm	NONDE	1.8	0.09
sarcoma	NONDE	0.6	0.584
germ cell neoplasm	NONDE	-1.2	0.242
normal solid tissue	DOWN	-3.3	0.002
blood neoplasm cell line	DOWN	-8.2	1.00E-10
solid tissue non neoplastic disease	DOWN	-10	1.00E-10
non neoplastic cell line	DOWN	-15	1.00E-10
solid tissue neoplasm cell line	DOWN	-53	1.00E-10
