# Supplementary material for: Inflammation Modulates RLIP76/RALBP1 Electrophile-Glutathione Conjugate Transporter and Housekeeping Genes in Human Blood-Brain Barrier Endothelial Cells
Source: PLoS One. 2015 Sep 25;10(9):e0139101. doi: 10.1371/journal.pone.0139101 (PMC4583384; doi:10.1371/journal.pone.0139101)
Supplement: S6 Table — Each metagroup contained at least 10 biological replicates. UP: up-regulated; DOWN: down-regulated; NONDE: non-differentially expressed. │t-statistic│ > 2 was found to be significant (p<0.05). (PDF) [file pone.0139101.s006.pdf]

Metagroup	Up/Down	t-statistic	p-value
acute myeloid leukemia	UP	22	$<1 \times 10^{-10}$
bronchoalveolar lavage cell	UP	20	$<1 \times 10^{-10}$
MCF7 breast epithelial adenocarcinoma	UP	20	$<1 \times 10^{-10}$
ht-29 colorectal adenocarcinoma	UP	15	$<1 \times 10^{-10}$
monocyte	UP	13	$<1 \times 10^{-10}$
macrophage	UP	12	$<1 \times 10^{-10}$
leukocyte	UP	11	$<1 \times 10^{-10}$
chronic myeloid leukemia	UP	9.7	$<1 \times 10^{-10}$
ovarian tumor	UP	9.5	$<1 \times 10^{-10}$
Hep-2 larynx sqamous cell carcinoma	UP	9.4	$<1 \times 10^{-10}$
hepatocellular carcinoma	UP	9	$<1 \times 10^{-10}$
A549 lung adenocarcinoma	UP	8.8	$<1 \times 10^{-10}$
breast cancer	UP	7.7	$<1 \times 10^{-10}$
MDA-MB-231 breast cancer	UP	7.3	$<1 \times 10^{-10}$
mononuclear cell infection	UP	7.1	$<1 \times 10^{-10}$
mcf-7aro breast epithelial adenocarcinoma	UP	6.3	1.42×10^{-9}
HeLa cervical adenocarcinoma	UP	6.2	1.61×10^{-9}
bladder cancer	UP	6.1	3.4×10^{-9}
embryonal rhabdomyosarcoma	UP	5.9	1.02×10^{-8}
MDA468 breast cancer	UP	5.4	2.41×10^{-7}
ssMCF7 breast cancer	UP	5.2	5.3×10^{-7}
B-cell lymphoma	UP	5.1	9.79×10^{-7}
embryonic skin fibroblast	UP	5	1.36×10^{-6}
primary intervertebral disc	UP	4.8	3.81×10^{-6}
mononuclear cell	UP	4.5	1.33×10^{-5}
RKO colon carcinoma	UP	4.3	3.33×10^{-5}
placenta basal plate	UP	4.2	5.23×10^{-5}
INCaP prostate cancer	UP	4	1.24×10^{-4}
ewings sarcoma	UP	4	1.38×10^{-4}
BT474 breast cancer	UP	3.9	1.73×10^{-4}
fetal lung fibroblast	UP	3.9	2.32×10^{-4}
universal reference	UP	3.6	5.22×10^{-4}
thyroid adenocarcinoma	UP	3.3	0.002
myelogenous leukemia	UP	3.3	0.002
k562 myelogenous leukaemia	UP	3.1	0.004
unknown lung adenocarcinoma	UP	2.9	0.006
uterine tumor	UP	2.9	0.007
IB3-1 adenovirus transformed bronchial epithelia	NONDE	2	0.066
myometrium	NONDE	1.8	0.088
brain tumor	NONDE	1.7	0.108

Metagroup	Up/Down	t-statistic	p-value
HeLa cervical adenocarcinoma transfected	NONDE	1.7	0.109
B cell	NONDE	1.7	0.121
acute promyelocytic leukemia	NONDE	1.6	0.137
T47D breast ductal carcinoma	NONDE	1.4	0.209
thymocyte	NONDE	1.3	0.233
mesenchymal stem cell	NONDE	1.1	0.311
hl60 promyelocytic leukemia	NONDE	0.9	0.426
ts anaplastic large cell lymphoma	NONDE	0.9	0.426
umbilical vein endothelial cell	NONDE	0.87	0.439
pc3 prostate cancer	NONDE	0.66	0.566
neuroblastoma	NONDE	0.5	0.668
lung cancer	NONDE	0.43	0.715
prostate cancer	NONDE	0.28	0.813
smooth muscle	NONDE	0.28	0.814
embryonic lung fibroblast	NONDE	0.2	0.864
renal cell carcinoma	NONDE	0.17	0.888
Kaposi sarcoma	NONDE	-0.67	0.555
preadipocytes	NONDE	-0.96	0.394
Calu-3 lung adenocarcinoma	NONDE	-1	0.365
oral squamous cell carcinoma	NONDE	-1.2	0.278
FM9514 human embryonic myoblast	NONDE	-1.3	0.246
precursor T lymphoblastic leukemia	NONDE	-1.7	0.124
blood	NONDE	-2	0.061
germ cell tumor	DOWN	-2.2	0.041
tonsil	DOWN	-2.4	0.023
skmel5 melanoma	DOWN	-2.6	0.017
prostate gland	DOWN	-2.7	0.01
kidney	DOWN	-2.7	0.013
colorectal cancer	DOWN	-3.6	6.85×10^{-4}
unknown lung small cell cancer	DOWN	-3.8	2.7×10^{-4}
lymph node	DOWN	-4.1	9.35×10^{-5}
epidermis dermatitis	DOWN	-4.2	5.57×10^{-5}
MOLT4 T cell acute lymphoblastic leukemia	DOWN	-4.3	4.45×10^{-5}
T cell diseased	DOWN	-5.5	1.03×10^{-7}
lymphocyte	DOWN	-5.5	1.53×10^{-7}
HEK293 embryonic kidney	DOWN	-5.7	5.06×10^{-8}
hematopoietic stem cell	DOWN	-6.1	3.06×10^{-9}
T cell	DOWN	-6.2	2.1×10^{-9}
caco2 colon adenocarcinoma	DOWN	-6.5	3.99×10^{-10}
hypothalamus	DOWN	-6.7	1.02×10^{-10}
CD138+ plasma cell myeloma	DOWN	-8	$<1 \times 10^{-10}$
heart	DOWN	-8.2	$<1 \times 10^{-10}$

Metagroup	Up/Down	t-statistic	p-value
heart disease	DOWN	-8.3	$<1 \times 10^{-10}$
skeletal muscle muscular dystrophy	DOWN	-10	$<1 \times 10^{-10}$
skeletal muscle	DOWN	-11	$<1 \times 10^{-10}$
brain	DOWN	-12	$<1 \times 10^{-10}$
caudate nucleus Huntingtons	DOWN	-12	$<1 \times 10^{-10}$
caudate nucleus	DOWN	-12	$<1 \times 10^{-10}$
brain bipolar disorder	DOWN	-12	$<1 \times 10^{-10}$
skeletal muscle diseased	DOWN	-12	$<1 \times 10^{-10}$
bronchial epithelia	DOWN	-13	$<1 \times 10^{-10}$
acute lymphoblastic leukemia	DOWN	-15	$<1 \times 10^{-10}$
frontal cortex Huntingtons	DOWN	-16	$<1 \times 10^{-10}$
frontal cortex	DOWN	-16	$<1 \times 10^{-10}$
cerebellum	DOWN	-19	$<1 \times 10^{-10}$
cerebellum Huntingtons	DOWN	-23	$<1 \times 10^{-10}$
