# Supplementary material for: Inflammation Modulates RLIP76/RALBP1 Electrophile-Glutathione Conjugate Transporter and Housekeeping Genes in Human Blood-Brain Barrier Endothelial Cells
Source: PLoS One. 2015 Sep 25;10(9):e0139101. doi: 10.1371/journal.pone.0139101 (PMC4583384; doi:10.1371/journal.pone.0139101)
Supplement: S7 Table — Each metagroup contained at least 10 biological replicates. UP: up-regulated; DOWN: down-regulated; NONDE: non-differentially expressed. │t-statistic│ > 2 was found to be significant (p<0.05). (PDF) [file pone.0139101.s007.pdf]

Metagroup	Up/Down	t-statistic	p-value
MCF7 breast epithelial adenocarcinoma	UP	27	$<1 \times 10^{-10}$
A549 lung adenocarcinoma	UP	17	$<1 \times 10^{-10}$
MDA-MB-231 breast cancer	UP	15	$<1 \times 10^{-10}$
HeLa cervical adenocarcinoma	UP	13	$<1 \times 10^{-10}$
lymphocyte	UP	12	$<1 \times 10^{-10}$
RKO colon carcinoma	UP	10	$<1 \times 10^{-10}$
ts anaplastic large cell lymphoma	UP	9.9	$<1 \times 10^{-10}$
Hep-2 larynx sqamous cell carcinoma	UP	9.9	$<1 \times 10^{-10}$
skeletal muscle diseased	UP	9.4	$<1 \times 10^{-10}$
Calu-3 lung adenocarcinoma	UP	9	$<1 \times 10^{-10}$
macrophage	UP	7.8	$<1 \times 10^{-10}$
unknown lung adenocarcinoma	UP	7.5	$<1 \times 10^{-10}$
embryonic lung fibroblast	UP	7.4	$<1 \times 10^{-10}$
mcf-7aro breast epithelial adenocarcinoma	UP	6.5	3.75×10^{-10}
ssMCF7 breast cancer	UP	6.4	7.57×10^{-10}
embryonic skin fibroblast	UP	6.1	4.02×10^{-9}
fetal lung fibroblast	UP	6.1	4.81×10^{-9}
FM9514 human embryonic myoblast	UP	5.8	2.58×10^{-8}
pc3 prostate cancer	UP	5.6	5.46×10^{-8}
IB3-1 adenovirus transformed bronchial epithelia	UP	5.6	6.5×10^{-8}
HeLa cervical adenocarcinoma transfected	UP	5.4	2.08×10^{-7}
T47D breast ductal carcinoma	UP	5.3	4.1×10^{-7}
ht-29 colorectal adenocarcinoma	UP	4.8	4.38×10^{-6}
skmel5 melanoma	UP	4.5	1.76×10^{-5}
HEK293 embryonic kidney	UP	4.3	3.24×10^{-5}
B cell	UP	4.3	3.38×10^{-5}
caco2 colon adenocarcinoma	UP	4.2	7.02×10^{-5}
skeletal muscle	UP	4.1	8.49×10^{-5}
MOLT4 T cell acute lymphoblastic leukemia	UP	4	1.17×10^{-4}
mesenchymal stem cell	UP	4	1.32×10^{-4}
umbilical vein endothelial cell	UP	3.9	1.96×10^{-4}
universal reference	UP	3.9	2.08×10^{-4}
BT474 breast cancer	UP	3.5	7.58×10^{-4}
Kaposi sarcoma	UP	3.4	0.001
MDA468 breast cancer	UP	3.2	0.002
unknown lung small cell cancer	UP	3.2	0.002
thymocyte	UP	3	0.004
INCaP prostate cancer	UP	3	0.005
skeletal muscle muscular dystrophy	UP	2.7	0.013
smooth muscle	UP	2.6	0.014

Metagroup	Up/Down	t-statistic	p-value
hematopoietic stem cell	UP	2.2	0.039
germ cell tumor	UP	2.1	0.049
preadipocytes	NONDE	1.8	0.096
k562 myelogenous leukaemia	NONDE	0.8	0.478
precursor T lymphoblastic leukemia	NONDE	0.73	0.521
monocyte	NONDE	0.47	0.686
epidermis dermatitis	NONDE	0.25	0.832
ewings sarcoma	NONDE	0.24	0.84
neuroblastoma	NONDE	-0.036	0.976
heart disease	NONDE	-0.38	0.745
placenta basal plate	NONDE	-0.73	0.519
tonsil	NONDE	-0.81	0.475
acute promyelocytic leukemia	NONDE	-1.4	0.219
embryonal rhabdomyosarcoma	NONDE	-1.7	0.109
oral squamous cell carcinoma	DOWN	-2.1	0.047
thyroid adenocarcinoma	NONDE	-2.1	0.052
B-cell lymphoma	NONDE	-2.1	0.056
uterine tumor	DOWN	-2.2	0.037
heart	DOWN	-2.2	0.038
myometrium	DOWN	-2.2	0.041
primary intervertebral disc	DOWN	-2.5	0.019
brain	DOWN	-2.9	0.006
hepatocellular carcinoma	DOWN	-3.1	0.004
bronchoalveolar lavage cell	DOWN	-3.2	0.003
myelogenous leukemia	DOWN	-3.2	0.003
renal cell carcinoma	DOWN	-3.3	0.002
brain tumor	DOWN	-3.5	0.001
bladder cancer	DOWN	-4.2	5.65×10^{-5}
prostate gland	DOWN	-4.3	4.4×10^{-5}
lymph node	DOWN	-5.1	1.19×10^{-6}
lung cancer	DOWN	-5.2	4.85×10^{-7}
prostate cancer	DOWN	-5.7	3.66×10^{-8}
CD138+ plasma cell myeloma	DOWN	-6	8.65×10^{-9}
brain bipolar disorder	DOWN	-6.1	4.6×10^{-9}
acute lymphoblastic leukemia	DOWN	-6.9	$<1 \times 10^{-10}$
kidney	DOWN	-7.7	$<1 \times 10^{-10}$
caudate nucleus	DOWN	-8.3	$<1 \times 10^{-10}$
frontal cortex	DOWN	-8.7	$<1 \times 10^{-10}$
T cell diseased	DOWN	-9	$<1 \times 10^{-10}$
frontal cortex Huntingtons	DOWN	-10	$<1 \times 10^{-10}$
bronchial epithelia	DOWN	-10	$<1 \times 10^{-10}$
hypothalamus	DOWN	-10	$<1 \times 10^{-10}$

Metagroup	Up/Down	t-statistic	p-value
T cell	DOWN	-11	$<1 \times 10^{-10}$
ovarian tumor	DOWN	-12	$<1 \times 10^{-10}$
cerebellum	DOWN	-12	$<1 \times 10^{-10}$
hl60 promyelocytic leukemia	DOWN	-12	$<1 \times 10^{-10}$
colorectal cancer	DOWN	-14	$<1 \times 10^{-10}$
caudate nucleus Huntingtons	DOWN	-14	$<1 \times 10^{-10}$
mononuclear cell	DOWN	-17	$<1 \times 10^{-10}$
cerebellum Huntingtons	DOWN	-17	$<1 \times 10^{-10}$
leukocyte	DOWN	-19	$<1 \times 10^{-10}$
mononuclear cell infection	DOWN	-20	$<1 \times 10^{-10}$
chronic myeloid leukemia	DOWN	-21	$<1 \times 10^{-10}$
blood	DOWN	-21	$<1 \times 10^{-10}$
breast cancer	DOWN	-24	$<1 \times 10^{-10}$
acute myeloid leukemia	DOWN	-38	$<1 \times 10^{-10}$
